# PPAR Beta/Delta and the Hallmarks of Cancer

**DOI:** 10.3390/cells9051133

**Published:** 2020-05-04

**Authors:** Nicole Wagner, Kay-Dietrich Wagner

**Affiliations:** Université Côte d’Azur, CNRS, INSERM, iBV, 06107 Nice, France; kwagner@unice.fr

**Keywords:** peroxisome proliferator-activated receptor, angiogenesis, proliferation, metastasis, immortality, resistance to cell death, growth suppressors, immune system, cellular metabolism

## Abstract

Peroxisome proliferator-activated receptors (PPARs) belong to the nuclear hormone receptor family. Three different isoforms, PPAR alpha, PPAR beta/delta and PPAR gamma have been identified. They all form heterodimers with retinoic X receptors to activate or repress downstream target genes dependent on the presence/absence of ligands and coactivators or corepressors. PPARs differ in their tissue expression profile, ligands and specific agonists and antagonists. PPARs attract attention as potential therapeutic targets for a variety of diseases. PPAR alpha and gamma agonists are in clinical use for the treatment of dyslipidemias and diabetes. For both receptors, several clinical trials as potential therapeutic targets for cancer are ongoing. In contrast, PPAR beta/delta has been suggested as a therapeutic target for metabolic syndrome. However, potential risks in the settings of cancer are less clear. A variety of studies have investigated PPAR beta/delta expression or activation/inhibition in different cancer cell models in vitro, but the relevance for cancer growth in vivo is less well documented and controversial. In this review, we summarize critically the knowledge of PPAR beta/delta functions for the different hallmarks of cancer biological capabilities, which interplay to determine cancer growth.

## 1. Introduction

Peroxisome proliferator-activated receptors (PPARs) belong to the group of nuclear receptors. They exist in three different isoforms: PPARα (NR1C1), PPARβ/δ (NR1C2) and PPARγ (NR1C3). They heterodimerize with RXR; and upon ligand binding act mainly as transcriptional regulators of specific target genes. Dependent on the tissue distribution, cofactors and availability of ligands, PPARs exert multiple functions (reviewed in [1]). PPARα is mainly expressed in liver, heart, brown adipose tissue, kidney and intestine and regulates energy homeostasis by activation of fatty acid catabolism and stimulation of gluconeogenesis [2]. PPARβ/δ is more or less ubiquitously expressed with some species differences, while PPARγ is expressed in white and brown adipose tissue, the gut and immune cells [1]. Endogenous ligands for PPARs are fatty acids, triglycerides, prostacyclins, prostaglandins and probably retinoic acid. Although varies different binding sites for PPARs in target genes have been reported, they share in general as a response element a direct repeat of the sequence AGGTCA, spaced by a single nucleotide, which was originally identified for PPARα (reviewed in [1]). Thus, in case more than one of the receptors is expressed in a certain cell-type, one could expect cross talk in response to endogenous or pan-PPAR pharmacological agonists. Specific agonists for PPARα are used classically for the treatment of dyslipidemia and agonists for PPARγ are insulin sensitizers to treat patients with type 2 diabetes. Currently, no PPARβ/δ activators or antagonists are in official clinical use. A recent review summarized novel developments regarding patents for PPAR modulators and possible novel clinical indications [3]. Clinical evidence for the use of PPAR agonists and antagonists is reviewed in [4]. Toxicological aspects and side effects of PPAR modulators have been reviewed recently [5]. Increasing interest focuses on potential implications of PPARs in cancer. The major clinical trials database (https://clinicaltrials.gov) lists one clinical trial for a PPARα antagonist for treatment of multiple kinds of cancer, 24 trials for modulators of PPARγ for cancer treatment, but none for PPARβ/δ. The human protein atlas (https://www.proteinatlas.org/ENSG00000112033-PPARD/pathology) lists low cancer type specificity, but detection of PPARβ/δ in all cancer types. A current major limitation for the investigation of PPARβ/δ expression in human cancer samples compared to healthy tissues is the quality of commercially available antibodies. In agreement with this, large differences for PPARβ/δ RNA and protein levels in tumors are noted in the human protein atlas. The protein expression is globally described, but not annotated to certain cell types in the different tumors. Correlations of tumor PPARβ/δ expression with patients outcome have been reviewed recently [6].

Earlier experimental results concerning the role of PPARβ/δ activation for cancer growth were completely controversial with one study showing that pharmacological activation with GW501516 enhanced tumor growth in Apc(min) mice [7], while another study in the same year in the same journal showed enhanced tumor growth in Apc(min) mice crossed with PPARβ/δ knockout mice [8]. Many studies using different cell models have been published afterwards. Several aspects of PPARβ/δ function with relevance for cancer growth have been reviewed recently [1,5,6,9,10,11]. 

On a global view, tumor progression is determined by the interplay of cancer cell proliferation, angiogenesis, resisting cell death, evading growth suppressors, activating invasion and metastasis, enabling replicative immortality, deregulating cellular metabolism and avoiding immune destruction, which was defined by Hanahan and Weinberg as the didactic concept of the “hallmarks of cancer” [12,13]. We will follow here this concept and review the knowledge of PPARβ/δ function for the different hallmarks of cancer capabilities. 

## 2. PPARβ/δ and Cell Proliferation

Most published papers focused on tumor growth-promoting or tumor-inhibiting actions of PPARβ/δ. Unfortunately, only few manuscripts distinguished between direct effects on cell proliferation and secondary effects, which might affect tumor growth. Thus, for simplification, we will summarize in this chapter the published results on cell proliferation as well as on general tumor growth. Table 1 summarizes published effects of PPARβ/δ on cell proliferation and tumor growth.

It has been shown that the PPARβ/δ agonist GW501560 increased VEGF expression in tumor cell lines [16] and promoted tumorigenesis in Apc(Min/+) mice [7,16] linking tumor cell growth and angiogenesis in the gut. Genetical disruption of PPARβ/δ in colon epithelial cells resulted in a lower incidence of azoxymethane-induced colon tumors and reduced VEGF expression [22]. Unfortunately, vessel formation has not been analyzed in detail in these models [16]. In the same azoxymethane-induced colon tumor model, also reduced tumor growth in response to GW0742, which was abolished by PPARβ/δ knockout [23] and a general reduced colon tumor growth in PPARβ/δ knockout mice [8] have been reported. This discrepancy remains unexplained. PPARγ and PPARβ/δ activation of VEGF and cyclooxygenase-2 (COX2) was confirmed in in the colorectal tumor cell lines SW480 and HT29 [49]. Curiously, that opposite regarding cancer growth and VEGF regulation was described using the KM12C colon cancer cell line with silencing of PPARβ/δ in mouse xenograft models [24]. Whether this reflects an unusual behavior of this specialized cell line remains to be determined. The tumor promoting action of GW501516 in Apc mice was confirmed recently and extended by the findings that the PPARβ/δ antagonist GSK3787 suppressed tumorigenesis. PPARβ/δ expression was significantly higher in human colorectal cancers compared to adenomatous polyps and normal mucosa [50] and also in the malignant cells—invasive front versus their paired tumor centers and adenomas—and proinvasive pathways (connexin 43, PDGFRb, AKT1, EIF4G1 and CDK1) were upregulated in response to PPARβ/δ stimulation [17]. Again, the opposite result has also been published for human and mouse tumor samples [51], while another important report using human colorectal cancer samples confirmed high expression of PPARβ/δ and COX2, which was correlated with the incidence of liver metastasis and identified as significant independent prognostic factor [52]. In colitis-associated colon cancer mouse models, PPARβ/δ overexpression promoted tumorigenesis in mice [20] and increased IL-6 expression and STAT3 phosphorylation, whereas concomitant 15-Lipoxygenase-1 expression in colonic epithelial cells suppressed these effects [19]. In an elegant study using different mouse lines, Beyaz et al. showed that high fat diet (HFD) via activation of PPARβ/δ augments the numbers and function of intestinal stem and progenitor cells. Pharmacological activation of PPARβ/δ using GW501516 recapitulated the effects of HFD on these cells. PPARβ/δ activation in the setting of a loss of the APC tumor suppressor gene allowed stem and progenitor cells to initiate tumorigenesis [21], which is in agreement with the studies mentioned above.

Regarding mammary neoplasia, Yuan et al. [36] showed in transgenic animals that activation of PPARβ/δ in the mammary epithelium resulted in progressive histopathologic changes that culminated in the appearance of estrogen receptor- and progesterone receptor-positive and ErbB2-negative infiltrating ductal carcinomas after 12 months in transgenic animals, while treatment with GW501516 shortened the interval until tumor appearance to 5 months. Histologically, Ki-67 expression was increased demonstrating enhanced proliferation of the epithelial cells, and several metabolic changes were observed (see below). Additionally, in animals with 3-phosphoinositide-dependent kinase-1 (PDK1) overexpression in mammary epithelium, GW501516 accelerated tumorigenesis, which was more pronounced in mice with PDK1 overexpression [38]. This is in agreement with many other reports as PDK1 overexpression resulted in an increase in PPARβ/δ expression and profound metabolic changes. Furthermore, GW501516 increased PPARβ/δ and PDK1 expression in mammary tumors [40]. In MMTV-ErbB2/HER2 onco-mice, knockout of FABP5, which shuttles ligands from the cytosol to nuclear PPARβ/δ was sufficient to reduce mammary tumorigenesis highlighting the importance of this molecule and endogenous PPARβ/δ ligands for cancer growth [39]. On the molecular level, epidermal growth factor receptor ligands signal through the ERK and the phophatidylinositol-3-kinase cascades to activate the transcription factor NF-kappaB. NF-kappaB increases via direct transcriptional activation the expression of FABP5 in MCF-7 breast cancer cells, which stimulates proliferation [53]. In Cox-2 overexpressing mice, mammary tumorigenesis was increased, which could be reverted by crossing them with PPARβ/δ knockout mice [37]. In severely immunocompromised mice, MCF-7 breast cancer cells with overexpression of PPARβ/δ produced bigger tumors and more metastasis compared to wild-type cells. Treatment of MCF-7 cells with PPARβ/δ antagonists in culture reduced significantly the number of these cells [35,54].

Martín-Martín et al. showed an opposite result for prostate carcinoma. PPARβ/δ mRNA was downregulated in prostate cancer specimens compared to benign prostate hyperplasia samples; and prostate epithelium-specific knockout of PPARβ/δ increased cellularity. Additional supporting evidence was obtained by the generation of different overexpression or silencing clones from different human prostate cancer cell lines. Mechanistically, PPARβ/δ exerted its activity in a DNA binding-dependent and ligand-independent manner, which involved regulation of the secretory trefoil factor family member 1 [25]. To which extend the stable cell clones and mice exposed during the entire lifespan to the Cre corresponding to tumor development in humans in vivo remains to be determined. In contrast, silencing of PPARβ/δ in prostate cancer cell lines inhibited tumor cell proliferation and tumor growth, which was attributed to activation of the ABCA1 cholesterol transporter-Caveolin1-TGFβ receptor signaling axis [26]. A similar observation in prostate cancer cells was published by Morgan et al., identifying fatty acid binding protein 5 (FABP5) as a direct target gene of PPARβ/δ [27].

Overexpression of PPARβ/δ decreased the cell number in neuroblastoma NGP, but not in SK-N-BE(2) and IMR-32 cell clones. In xenograft models, PPARβ/δ overexpression reduced tumor growth in NGP cell clones, but to a lesser extent in SK-N-BE(2) and IMR-32 cell clones [29]. As the level of overexpression of PPARβ/δ was highest in NGP cells, it is difficult to judge whether the different outcome is due to the different cell lines used or a response to the different levels of PPARβ/δ overexpression. Whether the results correspond to neuroblastoma pathogenesis in vivo remained an open question. A comparable observation was published by the same group when using testicular embryonal carcinoma cell clones with PPARβ/δ overexpression and the agonist GW0742 [41].

In transgenic hepatitis B virus (HBV) mice, long term treatment with the PPARβ/δ agonist GW0742 reduced the number of hepatic tumor foci. Based on reduced expression of cyclin D1 and c-Myc, a reduction in tumor cell proliferation has been proposed [30]. In human hepatocellular carcinoma cell lines, GW501516 increased proliferation, while RNAi against PPARβ/δ inhibited cell growth. PPARβ/δ activation up-regulates the expression of cyclooxygenase (COX)-2, a rate-limiting enzyme for prostaglandin synthesis and tumor growth in hepatocellular cancer lines [31].

Chronic exposure to ultraviolet light (UV) induced PPARβ/δ activity in the skin of mice. Increased PPARβ/δ activity directly stimulated Src expression, increased Src kinase activity and enhanced the EGFR/Erk1/2 signaling pathway, resulting in increased epithelial-to-mesenchymal transition (EMT) marker expression. PPARβ/δ-null mice developed fewer and smaller skin tumors. Furthermore, topical application of the PPARβ/δ antagonist GSK0660 prevented UV-dependent Src stimulation; and the expression of PPARβ/δ positively correlated with the expression of SRC and EMT markers in human skin squamous cell carcinoma (SCC) highlighting the clinical relevance of these findings [42]. Another report claimed that the agonist GW0742 delayed chemical induced skin carcinogenesis; combination of GW0742 and the COX2 inhibitor nimesulide resulted in a further decrease of tumor multiplicity in wild-type mice, but not in PPARβ/δ-null mice [55]. Given that the graphs in the different groups for tumor incidence, multiplicity and size look comparable and no statistical information is provided, it is difficult to follow this line of evidence, which is in sharp contrast to many other published papers. Even more surprising, the same authors reported earlier for a comparable model no effect of GW0742 on chemical induced skin carcinogenesis [56], or no combined effects for GW0742 and the COX2 inhibitor nimesulide in induced colon cancers [57] or independence of the COX2 inhibitor effects on PPARβ/δ [58].

High PPARβ/δ expression was detected in human melanoma compared to normal skin [32]. PPARβ/δ activation using GW0742 or GW501516 inhibited proliferation of different melanoma cell lines [32,33], which was due to direct transcriptional repression of the Wilms’ tumor suppressor WT1 and its downstream target genes zyxin [59] and nestin [59,60,61].

In non-small cell lung cancer (NSCLC) cell lines, PPARβ/δ activation increased proliferation and survival, while PPARβ/δ knock-down reduced viability and increased apoptosis. As reported for colon cancer, PPARβ/δ agonists induced VEGF transcription in NSCLC cell lines. Furthermore, increased expression of PPARβ/δ and VEGF in human non-small cell lung cancer samples compared to normal lung tissues has been detected [43,62]. In contrast, a study using only two lung cancer cell lines in vitro, did not find any effects on cell proliferation in response to PPARβ/δ activation [44]. In a transgenic mouse model of RAF-induced lung adenoma, tumor growth in mice lacking one or both alleles of PPARβ/δ was reported to be increased [45]. However, the histological analysis performed in this model was superficial and statistical information lacking.

We showed in liposarcoma cell lines that PPARβ/δ activation increases proliferation, which is abolished by a PPARβ/δ–siRNA or a specific PPARβ/δ antagonist. These effects were mediated via direct transcriptional repression of leptin by PPARβ/δ. PPARβ/δ was highly expressed in liposarcoma compared to lipoma and correlated with increased proliferation in human tumor samples [46].

PPARβ/δ was increased in benign and malignant human thyroid tumors and correlated with the proliferation marker Ki67. Overexpression of PPARβ/δ in thyroid cells and treatment with GW501516 increased cell proliferation in a cyclin E1-dependent manner. Specificity of the findings was proven by reduction of cyclin E1 expression and cell proliferation in response to RNAi against PPARβ/δ [47].

Epithelial ovarian cancer cell lines expressed high levels of PPARβ/δ. Inhibition of PPARβ/δ reduced epithelial ovarian cancer cell proliferation and reduced tumor growth in vivo. Mechanistically, aspirin, a nonsteroidal anti-inflammatory drug that preferentially inhibits COX-1, compromised PPARβ/δ function and cell growth by inhibiting extracellular signal-regulated kinases 1/2 [48].

Although still some controversies exist, PPARβ/δ expression has been documented in a broad variety of different tumor samples and cancer cell lines. In the majority of published reports, PPARβ/δ activation or overexpression was associated with increased cancer cell and tumor growth, some opposite results may be explained by use of different clonal cell lines or different genetic backgrounds and models in mice.

## 3. PPARβ/δ and Angiogenesis 

In contrast to PPARα and PPARγ, PPARβ/δ is a proangiogenic member of the PPAR family [63]. Vascular cell expression of PPARβ/δ has first been reported in the late 90s by Xin et al., 1999, using mRNA analysis [64] and Bishop-Bailey and Hla, 1999, employing Northern blot techniques [65]. In addition, PPARβ/δ expression in vascular smooth muscle cells had been observed by Bishop-Bailey in 2000 [66].

However, no specific functions of PPARβ/δ in the vasculature were discovered at that time, due to the lack of specific ligands. The first synthetic PPARβ/δ and PPARγ non-thiazolidinedione agonist L-165041 was established in 1999 [67], followed later by the highly selective PPARβ/δ agonists GW0742 and GW501516 [68].

A first report shading light on the function of PPARβ/δ in vascular cells appeared in 2001. Hatae and colleagues observed that prostacyclins induce apoptosis via PPARβ/δ activation in HEK293 cells whereas endothelial cells, which express cytoplasmic prostacyclin receptors are protected from apoptosis. They concluded that prostacyclin-dependent receptor activation results in increased cAMP levels in endothelial cells, which protects from apoptosis while direct prostacyclin activation of PPARβ/δ in cells lacking cytoplasmic prostacyclin receptors is proapoptotic [69]. A second investigation focusing on endothelial cell apoptosis demonstrated a protective action of L-165041 as well as of carbaprostacyclin (cPGL2) upon H_2_O_2_ induced apoptosis. Both substances increased expression of PPARβ/δ; knockdown of PPARβ/δ abrogated the apoptosis diminishing effects of both agents. As the molecular mechanism of this apoptosis protective function of PPARβ/δ in endothelial cells, the authors proposed the direct transcriptional activation of 14-3-3alpha protein, a cytosolic protein involved in apoptosis protection, by PPARβ/δ [70]. A later study further added activation of endothelial 14-3-3epsilon protein by PPARβ/δ agonists to the antiapoptotic role [71]. Non-steroidal anti-inflammatory drugs (NSAIDs) can induce endothelial cell apoptosis by disconcerting these transcriptional pathways [72].

PPARβ/δ agonists became of particular interest in vascular biology as they were shown to potently inhibit vascular inflammation and reduce atherosclerosis [73]. They inhibit tumor necrosis factor alpha (TNFα) mediated endothelial inflammation, evidenced by decreased expression of vascular cell adhesion molecule-1 (VCAM-1), monocyte chemotactic protein-1 (MCP-1) expression and inhibition of monocyte binding of TNFα stimulated endothelial cells treated with the PPARβ/δ agonist L-165041 [74]. It has been proposed that PPARβ/δ further controls inflammation via a ligand-dependent interaction with the transcriptional repressor BCL-6. In the absence of other ligands, PPARβ/δ binds BCL-6. When activated with a PPARβ/δ ligand, BCL-6 is released and can suppress proinflammatory pathways [65,75]. Later reports confirmed the anti-inflammatory effect of PPARβ/δ in endothelium [76,77]. PPARβ/δ also inhibits vascular smooth muscle inflammation by transcriptional activation of transforming growth factor (TGF)β1. The decreased MCP-1 expression induced by PPARβ/δ was shown to be mediated by the effector of TGF-β1, Smad3 [78].

Activation of PPARβ/δ has also been reported to prevent endothelial dysfunction by reducing oxidative stress [79]. In diabetic mice, PPARβ/δ activation mediated through phosphatidylinositol 3-kinase (PI3K) and Akt an increase of endothelial nitric oxide synthase (eNOS) activity and nitric oxide (NO) production and improved endothelium-dependent relaxation parameters [80]. In high glucose induced impairment of insulin signaling, PPARβ/δ activation restores endothelial function in part through pyruvate dehydrogenase kinase (PDK) 4 activation, thus preserving the insulin-Akt-eNOS pathway impaired by high glucose [81].

Despite its anti-inflammatory and anti-atherosclerotic functions in the vasculature, PPARβ/δ is a major factor for acute vascular hyperpermeability and vasodilatation, key features of allergic reactions, which can lead to lethal systemic anaphylaxis. The group of Michalik recently demonstrated that selective vessel-specific deletion of PPARβ/δ is sufficient to inhibit VEGF or IgE- induced acute vascular hyperpermeability and vasodilatation, most likely due to activity modulation of kinase pathways and destabilization of cell-to-cell adherens junctions. Inhibition of PPARβ/δ should be considered as a therapeutic approach in acute allergic and inflammatory diseases with disturbed endothelial integrity [82].

The first detailed report about the proangiogenic function of PPARβ/δ appeared in 2007. The selective PPARβ/δ ligand GW501516 was tested at this time in phase II clinical trials for the treatment of dyslipidemia. Using a variety of in vitro and ex vivo approaches, the authors clearly demonstrated that PPARβ/δ induces endothelial cell migration, proliferation and tube formation. They further described an increase of vascular endothelial growth factor (VEGF) expression upon activation of PPARβ/δ and already cautioned against possible negative side effects of agonist treatment in patients susceptible for “angiogenic diseases”, such as elderly persons prone to cancer incidence or diabetic individuals with retinopathies [83].

In vivo studies further showed that pharmacological activation with GW0742 as well as muscle specific transgenic overexpression of PPARβ/δ resulted in a rapid increase of capillary density and oxidative fiber numbers in skeletal muscle, resembling the muscular phenotype induced by regular physical training. It had been proposed that the observed effects were the calcineurin-nuclear factor of activated T cells (NFAT) pathway dependent, as inhibition of calcineurin by cyclosporine A (CsA) totally abolished the observed effects of pharmacological activation of PPARβ/δ [84]. Our group further demonstrated the function of PPARβ/δ in physiological vascularization. Treatment of mice with the agonist GW0742 resulted in rapid cardiac growth and vascularization without functional impairment as reflected by normal echocardiographic parameters. The cardiac hypertrophy accompanied by intensive vascularization resembled the cardiac phenotype obtained by long-term voluntary exercise. As the underlying molecular mechanism of this PPARβ/δ action, we identified the calcineurin-nuclear factor of activated T cells (NFAT) pathway [85]. However, it was unclear if the observed increased vascularization was a secondary effect of the myocardial hypertrophy or if the induction of cardiac growth was due to the increased angiogenesis. We therefore generated conditional mice with inducible vessel specific overexpression of PPARβ/δ and observed that vascular overexpression of PPARβ/δ was sufficient to induce a rapid cardiac hypertrophy. Nevertheless, the increased angiogenesis did not ameliorate cardiac function after myocardial infarction [86]. Similar observations were made using pharmacological activation of PPARβ/δ after myocardial infarction; also in this setting the increase in angiogenesis did not ameliorate the clinical outcome [87]. The proangiogenic function of PPARβ/δ was also exploited in other therapeutic approaches in ischemic cardiovascular diseases. Bone marrow derived endothelial progenitor cells (EPCs) represent an interesting path in the therapy of ischemic diseases, but due to their low number their clinical use is limited. Han and colleagues investigated the effects of PPARβ/δ agonists GW501516 or L-165041 on EPCs and found an increase of angiogenic EPC properties including increased migration, proliferation and tube formation in response to activation of PPARβ/δ. These effects were phosphatidylinositol 3-kinase/Akt pathway dependent. Systemic administration of PPARβ/δ agonists led to an increase of hematopoietic stem cells in bone marrow and blood as well as to an enhanced vascularization in ischemic hindlimb models and corneal neovascularization in vivo [88].

The therapeutic potential of PPARβ/δ modulation on aspects of ocular neovascularization, a common feature of premature or diabetic retinopathy, as well as age-related macular degeneration, the leading causes of irreversible blindness, was studied using human retinal microvascular endothelial cells (HRMEC) and in vivo models of oxygen-induced retinopathy (OIR). The authors demonstrated a stimulation of ocular vascularization with PPARβ/δ activation. Furthermore, using the selective PPARβ/δ antagonist GSK0660 [89], the potential therapeutic utility of PPARβ/δ inhibition was proven. GSK0660 decreased HRMEC migration, proliferation, and tube formation and neovascularization in OIR [90].

The effects of PPARβ/δ on tumor angiogenesis were first investigated in 2007. Employing B16 melanoma and LLC1 (Lewis lung carcinoma) tumor cell inoculated in PPARβ/δ^−/−^ mice, Müller-Brüsselbach and colleagues demonstrated cancer vascularization defects and diminished tumor blood flow, resulting in reduced tumor growth in animals lacking PPARβ/δ. In contrast to the report from Piqueras and colleagues [83], the authors observed a hyperproliferative state of endothelial cells, leading to the formation of immature and dysfunctional microvessels upon deletion of PPARβ/δ. On a molecular level, decreased expression of the antiproliferative cyclin-dependent kinase inhibitor 1C (Cdkn1c) was observed in PPARβ/δ^−/−^ cells isolated from Matrigel plugs, which might explain the proliferative immature state of PPARβ/δ^−/−^ endothelium [91].

However, in a second study from this group, diminished expression of chloride intracellular channel protein 4 (Clic4) and increased expression of cellular retinol binding protein 1 (Crbp1) were observed in PPARβ/δ^−/−^ fibroblasts and endothelial cells as compared to wildtype cells [92]. Clic4 promotes endothelial cell proliferation, capillary network and lumen formation [93], whereas Crbp1 binding retinoids in contrast favors growth arrest and differentiation [94]. This is in discrepancy to the observed hyperproliferative state of PPARβ/δ^−/−^ endothelial cells observed by the group of Müller-Brüsselbach [91] and fits to the conclusions made by Piqueras and colleagues that PPARβ/δ stimulates endothelial cell proliferation [83].

An important study further confirmed the strong implication of PPARβ/δ in proangiogenic stimulation favoring tumor progression. Abdollahi and coworkers aimed to identify genes involved in the “angiogenic switch”, the shift of an angiogenic balance to a proangiogenic state, one hallmark of cancer progression. Human microvascular cells were submitted to proangiogenic stimuli and subsequent cDNA arrays performed to identify differentially expressed genes upon proangiogenic stimulation. Further selection of genes based on their involvement in the angiogenic network identified PPARβ/δ as a “hubnode” in the “angiogenic switch”. The authors confirmed their findings in vivo using B16 melanoma and LLC1 Lewis lung carcinoma inoculated in PPARβ/δ^−/−^ mice, in which they observed dramatically reduced tumor angiogenesis and growth. PPARβ/δ expression levels in human cancer samples further correlated with advanced stages of tumor progression and metastasis [95,96].

Recently, PPARβ/δ activators (L165041 and GW501516) were shown to induce interleukin 8 (Il-8) expression in endothelial cells by transcriptional and posttranscriptional mechanisms [97], and enhanced production of IL-8 due to PPARβ/δ activation caused not only elevated tumor angiogenesis, but also metastasis formation in vivo [98].

Our group further confirmed the general tumor-angiogenesis and cancer growth promoting effect of PPARβ/δ [14]. Although we observed a decrease of LLC1 cancer cell proliferation in vitro upon treatment with GW0742, tumor growth and metastases formation in LLC1 cancer bearing animals was enhanced upon administration of the PPARβ/δ agonist. Tumor vascularization was strongly increased, which supports the hypothesis that enhancement of angiogenesis by PPARβ/δ dominates the eventually growth-inhibiting function on cancer cells. To further determine the functional relevance of PPARβ/δ for tumor vascularization and identify angiogenic signaling pathways, we made use of mice with conditional inducible vascular overexpression of PPARβ/δ subcutaneously implanted with LLC1 cells. Vessel-specific overexpression of PPARβ/δ was sufficient to increase cancer growth, progression and metastases formation. Tumor-sorted endothelial cells were submitted to RNA-sequencing; 283 genes were found to be differentially expressed and cluster analysis revealed mostly up-regulation of genes upon overexpression of PPARβ/δ in endothelial cells. This argues for an angiogenesis boosting effect of PPARβ/δ rather than a repression of antiangiogenic molecules to enhance angiogenesis. We identified six potential target genes of PPARβ/δ, all of them known to be involved in tumor angiogenesis, by combining the top ten network analysis with a search for PPAR responsive elements: Vegf receptors 1 (Flt1), 2 (Kdr) and 3 (Flt4), [99,100], and platelet-derived growth factor receptor beta (Pdgfrβ) [101], platelet-derived growth factor subunit B (Pdgfb) [102] and the tyrosinkinase KIT c-kit [103,104]. Finally, we confirmed that PPARβ/δ directly transcriptional activates Pdgfrβ, Pdgfb, and c-Kit. PPARβ/δ tumor-angiogenesis promoting effects are mediated via activation of the PDGF/PDGFR pathway, c-Kit and probably the VEGF/VEGFR pathway [14]. 

Despite their beneficial effects on vascular inflammation and atherosclerosis, the therapeutic use of PPARβ/δ agonists could be critical in cancer patients and should therefore in general not be considered as a therapeutic option.

## 4. PPARβ/δ and Cell Death

The first study demonstrating an inhibitory function of PPARβ/δ in cancer cell death appeared in 1999. He and colleagues revealed that the adenomatous polyposis coli (APC) tumor suppressor represses PPARβ/δ expression through inhibition of β-catenin/Tcf-4 regulated transcription (CRT). APC/β-catenin mutations can therefore lead to increased PPARβ/δ activity. Nonsteroidal anti-inflammatory drugs (NSAIDs) Sulindac and Indomethacin promoted apoptosis of colorectal cancer cells, which could be inhibited by overexpression of PPARβ/δ. The authors demonstrated that NSAIDs suppressed activity of PPARβ/δ through the direct inhibition of DNA binding activity. As fatty acids and eicosanoids are ligands and modifiers of PPAR activity, NSAID-dependent changes in eicosanoid metabolism could also contribute to inhibition of PPARβ/δ activity. NSAIDs were therefore considered as an important therapeutic approach in colorectal carcinoma as they inhibited apoptosis-preventing PPARβ/δ activity also in the context of frequently occurring APC/β-catenin mutations [105]. Other groups demonstrated that cyclooxygenase-derived prostaglandin E2 (PGE2) inhibits colon cancer cell apoptosis through the indirect transactivation of PPARβ/δ. Of note, the authors showed that PGE2 specifically regulates PPARβ/δ, not the other PPARs. The apoptosis inhibiting effects of PGE2 are mediated through indirect mediation of PPARβ/δ by activation of the PI3K/Akt signaling pathway [106]. Gupta et al. confirmed the antiapoptotic effect of PPARβ/δ activation in wildtype and PPARβ/δ-deficient HCT116 colon carcinoma cells. Pretreatment of wildtype HCT116 cells with GW501516 reduced serum withdrawal induced apoptosis, which was not the case in PPARβ/δ-deficient HCT116 cells, suggesting a specific effect of PPARβ/δ activation [7]. Nonsteroidal anti-inflammatory drugs (NSAIDs) were also shown to induce colorectal cancer cell apoptosis through other PPARβ/δ mediated mechanisms. NSAIDs inhibited 14-3-3ε protein expression, leading to apoptosis, accompanied by a decrease of cytosolic and an increase of mitochondrial Bad [107]. The authors had already shown that PPARβ/δ transcriptionally activates 14-3-3ε [70], and further confirmed their hypothesis in this study by overexpression of PPARβ/δ, which rescued colorectal cancer cells from NSAID induced apoptosis and upregulated 14-3-3ε protein levels. This additionally implicates the PPARβ/δ 14-3-3ε pathway in colon cancer cell survival [107]. Again, in the setting of colorectal cancer, it has been shown that PPARβ/δ overexpression or activation antagonizes PPARγ-induced apoptosis of cancer cells. PPARγ agonists induce apoptosis in these cancer cells through reduction of survivin, which in turn leads to apoptosis through increased caspase-3 activity. PPARβ/δ agonists inhibit induction of this apoptotic pathway by increasing survivin expression levels [108]. The apoptosis inducing effects of NSAIDs in colon cancer were also linked to 15-lipoxygenase-1 (15-LOX-1) upregulation. 13-S-hydroxyoctadecadienoic acid (13-S-HODE), the primary product of 15-LOX-1 metabolism of linoleic acid, was found to decrease activity and downregulate expression of PPARβ/δ in colon cancer cells, thereby inducing apoptosis [109]. An interesting study of Cutler and colleagues showed that fibroblasts isolated from the mucosa of hereditary non polyposis colorectal cancer (HNPCC) patients produced 50-fold more PGE2 than normal fibroblasts [110]. PGE2 inhibits apoptosis of colonic carcinoma cells through the activation of PPARβ/δ [106]. As HNPCC patients are more susceptible to develop colorectal cancer (CRC), the authors hypothesized that the overproduction of PGI2 from the stroma of HNPCC patients prevents apoptosis of neoplastic lesions through activation of PPARβ/δ and therefore facilitates progression into a malignant state of CRC [110]. In contrast to all these studies, indicating an antiapoptotic function of PPARβ/δ in colon cancer cells, one report suggested a proapoptotic function of PPARβ/δ in the setting of colon carcinoma. In a model of chemically induced colon carcinogenesis using wildtype and PPARβ/δ knockout mice, treatment of mice with the agonist GW0742 resulted in higher colonic cell apoptosis in wildtype animals as assessed by TUNEL staining and subsequent quantification of cell counts from colon sections, which does not really assure cancer cell specificity. No changes in apoptotic cell counts were observed in colons from PPARβ/δ knockout mice upon agonistic activation of PPARβ/δ [23].

Maggiora et al. investigated the effects of linoleic (LA) and conjugated-linoleic acids (CLA) on the growth of several human tumor cell lines, comprising prostate, bladder, liver, glioblastoma and breast cancer cells. In contrast to Las, CLAs had a strong growth inhibitory effect in the cancer cell lines tested and were able to induce apoptosis in the more deviated cells. PPARβ/δ levels decreased strongly in apoptotic cancer cells upon CLA treatment, but not in cell lines where only an inhibition of cell proliferation without subsequent cell death could be observed [111].

Our group investigated the effects of PPARβ/δ activation on human and mouse melanoma cells. Although we could observe a reduction of melanoma cell proliferation upon PPARβ/δ activation either with GW0742 or with GW501516 at nanomolar concentrations, we did not observe changes in melanoma cell apoptosis [32].

In one lung cancer cell line, PPARβ/δ activation with the agonist L165041 or treatment with the NSAID Indomethacin alone had no effect on apoptosis, however, a combination of these molecules induced apoptosis in this cancer cell line [112]. In contrast, activation of PPARβ/δ with the more specific agonist GW501516 has been demonstrated to inhibit cisplatin-induced apoptosis in different lung cancer cell lines [62]. In line with this latter finding, Genini et al. reported enhanced apoptosis in different human non-small cell lung cancer (NSCLC) lines upon knockdown of PPARβ/δ [43]. We investigated the effects of PPARβ/δ activation or antagonism on mouse Lewis lung carcinoma cells and observed no differences in apoptosis for neither modulation of PPARβ/δ activity [14].

In contrast to the studies mentioned above, which mostly describe an antiapoptotic function of PPARβ/δ in cancer cells, Foreman and colleagues postulated a proapoptotic action of PPARβ/δ in a mouse mammary gland cell line. Treatment with very high concentrations of the PPARβ/δ agonist GW501516 (10 µmolar) for 24 h increased early apoptosis in this cell line, as analyzed by annexin V staining. However, prolonged treatment for 48 h at the same concentrations had no effect on apoptosis, which could raise some doubts concerning the conclusions given in this study [113]. A study from the same group could neither confirm the observation that NSAIDs decrease PPAR activation and expression in colon cancer cells, nor that PPARβ/δ exerts an antiapoptotic function in the setting of colon cancer. Using different human colon cancer cell lines treated with hydrogen peroxide to induce apoptosis and NSAIDs and different concentrations of the PPARβ/δ agonist GW0742, the authors did not observe a decrease of early (evidenced by annexin V labeling) or late (analyzed by PARP cleavage) apoptosis upon PPARβ/δ activation [51]. Bell and colleagues demonstrated that inhibition of PPARβ/δ using siRNA mediated knockdown or the antagonist GSK0660 sensitized neuroblastoma cells to all-trans retinoic acid induced cell death [114]. In line with this proapoptotic function of PPARβ/δ, Péchery and colleagues reported enhanced apoptosis in tumor cells derived from high-grade bladder tumor upon activation with the PPARβ/δ agonist GW501516 [115]. Using only one prostate cancer cell line it has been postulated that the inhibition of PPARβ/δ with the antagonist GSK0660 partially inhibited ginsenoside Rh2 induced apoptosis [116]. In line with this study, another group recently reported a proapoptotic role of PPARβ/δ in prostate cancer cells. Treatment of one prostate cancer cell line with Telmisartan, an angiotensin receptor blocker, induced apoptosis, which could be partially inhibited by pharmacological or genetic down-regulation of PPARβ/δ activity or expression [117]. Additionally, in a nasopharyngeal carcinoma cell line, proapoptotic functions of PPARβ/δ could be demonstrated. Using in vitro and in vivo xenograft assays, high concentrations of GW501516 (10 or 30 µmolar) induced apoptosis of the nasopharyngeal cancer cells. The authors proposed as underlying mechanisms the activation of adenosine monophosphate-activated protein kinase (AMPKα) and downregulation of integrin-linked kinase (ILK), as the AMPK inhibitor compound C was able to inhibit the reduction of ILK expression induced by GW501516 [118]. Employing the same cell line, the authors further implicated the microRNA miR-206 in the apoptosis promoting effects of PPARβ/δ activation, as they observed an induction of miR-206 upon GW501516 mediated PPARβ/δ activation, which could be antagonized by the PPARβ/δ antagonist GSK3787 or the AMPK antagonist dorsomorphin [119].

In conclusion, it is not perfectly clear if PPARβ/δ prevents or stimulates cancer cell death. Although the majority of studies suggest that PPARβ/δ has an antiapoptotic function in cancer cells, some reports evoke the contrary and others do not observe implication of PPARβ/δ in apoptotic cancer cell death at all. This might be due to cancer cell type specific differences, but also to discrepancies in experimental set ups.

## 5. PPARβ/δ and Tumor Suppressors

In addition to positive regulation of growth-promoting signals, cancer development also requires inhibition of negative growth regulators, i.e., escaping the action of tumor suppressor genes [12]. Although a large number of publications described the overall effects of PPARβ/δ modulation on tumor growth, knowledge on PPARβ/δ and tumor suppressor genes is relatively limited. Mice with mutations in the adenomatous polyposis coli (APC) tumor suppressor are frequently used as a tool for PPAR research in colon cancer, but also a direct function of the APC tumor suppressor on PPARβ/δ expression has been described. APC represses PPARβ/δ expression through inhibition of β-catenin/Tcf-4 regulated transcription in colon cancer cells [105]. Besides colon cancer cells, inactivating mutations in APC or the Axin tumor suppressor proteins or activating mutations in β-catenin resulting in positive effects on T-cell factor (TCF)-regulated transcription have been described in several cancer types. Zhai et al. reported mutations leading to β-catenin deregulation in half of ovarian endometrioid adenocarcinomas. They found elevated expression of the MMP-7, CCND1 (Cyclin D1), CX43 (Connexin 43), ITF2 and also PPARβ/δ genes in ovarian endometrioid adenocarcinomas with deregulated β-catenin [120]. Transformation of intestinal epithelial cells with the K-Ras oncogene led to increased expression and activity of PPARβ/δ. Mechanistically, PPARβ/δ up-regulation was due to increased mitogen-activated protein kinase activity; and PPARβ/δ activation required the endogenous production of prostacyclins via the cyclooxygenase-2 pathway [121]. An initial important report from mice with inactivation of the APC tumor suppressor showed that treatment with the PPARβ/δ agonist GW501516 resulted in a significant increase in the number and size of intestinal polyps [7]. In contrast to the reports mentioned above, another study confirmed APC/beta-catenin-dependent expression of Cyclin D1, while expression of PPARβ/δ was not different in colon or intestinal polyps from wild-type or Apc(min) heterozygous mice or in human colon cancer cell lines with mutations in APC or beta-catenin [122]. This study based exclusively on the use of a polyclonal antibody in Western blots. The quality of the available PPARβ/δ antibodies is still a matter of concern.

Regarding the Wilms’ tumor suppressor WT1, we showed that PPARβ/δ activation in melanoma cells inhibits its expression via direct transcriptional repression [32]. WT1 was originally identified as a tumor suppressor based on its mutational inactivation in nephroblastoma [123,124], but later studies provided evidence that WT1 might act as an oncogene [60,101,104,125,126]. Wt1 was up-regulated instead of downregulated in endothelial cells with PPARβ/δ overexpression [14], which suggests cell-type dependent differential regulation of Wt1 by PPARβ/δ. Whether PPARβ/δ is a direct activator of WT1 in endothelial cells and other cell-types remains to be determined.

Epidermal growth factor receptor (EGFR) signaling promotes breast cancer cell proliferation and tumorigenesis. It has been shown that EGFR ligands signal through the ERK and the phophatidylinositol-3-kinase cascades, resulting in activation of the transcription factor NF-kappaB. The NF-kappaB transcription factor directly activates the promoter of fatty-acid binding protein 5 (FABP5) resulting in increased FABP5 protein expression, which in turn shuttles endogenous ligands to PPARβ/δ [53]. In contrast, Krüppel-like factor KLF2 inhibits FABP5 protein expression and subsequent PPARβ/δ activation and thus, might act as a tumor suppressor in breast cancer cells [53].

Transducer of ErbB-2.1 (Tob1) is another tumor suppressor protein, which is inactivated in different cancer types including gastrointestinal cancers. Overexpression of Tob1 in gastric cancer cell lines induced the expression of Smad4 and p15. Tob1 decreased the phosphorylation of Akt and glycogen synthase kinase-3β (GSK3β), resulting in reduced expression and the transcriptional activity of β-catenin, which in turn decreased the expression of PPARβ/δ, cyclin D1, cyclin-dependent kinase-4 (CDK4) and urokinase plasminogen activator receptor (uPAR) in gastric cancer cells [127]. These data are in agreement with the general regulation of PPARβ/δ by β-catenin and provide an additional complex signaling pathway for stimulation of PPARβ/δ activity in cancer progression.

In neuroblastoma cell lines, all-trans-retinoic acid reduced expression of the stem cell factor Sox2 in cell lines with low expression of the tumor suppressor p53, while this was not the case in cells with wild type p53. However, PPARβ/δ activation with GW0742 reduced SOX2 expression independent on the p53 status of the cells. The authors concluded that activating PPARβ/δ induces cell differentiation through p53- and SOX2-dependent signaling pathways in neuroblastoma cells and tumors [29]. However, the exact interaction between retinoic acid and PPARβ/δ signaling on SOX2 expression and the possible role of p53 therein remains to be determined.

In smooth muscle cells, the PPARβ/δ agonist L-165041 inhibited dose-dependently proliferation by blocking G(1) to S phase progression and repressing the phosphorylation of retinoblastoma protein (Rb). In a carotid artery injury model in vivo, L-165041 inhibited neointima formation [128]. To our knowledge, this is the only report linking retinoblastoma protein and PPARβ/δ activation. Whether these findings are relevant for cancer cell proliferation or tumor angiogenesis remains to be determined.

## 6. PPARβ/δ and Invasion and Metastasis

Abdollahi et al. were the first to correlate PPARβ/δ expression levels with advanced pathological tumor stage and increased risk for distant metastasis. Statistical analyses of PPARβ/δ expression in published large-scale microarray data from cancer patients with prostate, breast, and endometrial adenocarcinoma revealed significantly increased PPARβ/δ expression levels in cases of higher malignant grade and distant metastasis formation [95]. Similar observations were made by Yoshinaga and colleagues who found an increased risk for colorectal cancer patients with high expression of PPARβ/δ and cyclooxygenase (COX)2 in the primary tumor to develop distant liver metastasis, consequently leading to a poor prognostic outcome [96]. In contrast to these studies, one group reported decreased invasion capacity of pancreatic cancer cells in vitro upon PPARβ/δ activation with GW501516 as well as downregulated prometastatic Matrix metalloproteinase-9 (MMP9) expression [129]. A similar study implying in vitro approaches using breast cancer cell lines demonstrated decreased migration and invasion upon PPARβ/δ activation with GW501516. PPARβ/δ mediated inhibition of breast cancer cell migration and invasion was proposed to be regulated via thrombospondin-1 (TSP-1) and its degrading protease, a disintegrin and metalloprotease domains with thrombospondin motifs 1 (ADAMTS1), as knockdown of ADAMTS1 reduced the effects of PPARβ/δ activation; and ADAMTS1 promoter activity was increased by GW501516 [130].

Interestingly, yeast-two hybrid screening identified the metastasis suppressor NDP Kinase alpha (NM23-H2) as a binding protein of PPARβ/δ [131]. NM23 genes have been shown to suppress metastasis development [132]. Overexpression of NM23-H2 in cholangiocarcinoma cells downregulated PPARβ/δ expression, impedes PPARβ/δ promoter activity and diminishes GW501516 induced cholangiocarcinoma cell proliferation. Reactivation of NM23-H2 was suggested as a therapeutic approach in cholangiocarcinoma metastasis [131].

Zuo and collaborators further demonstrated the importance of PPARβ/δ in metastatic cancer. Using an experimental mouse model of metastasis formation by tail vein injection of syngenic tumor cells (B16 melanoma and LLC1 Lewis lung carcinoma cells), the authors showed that PPARβ/δ knockdown in the respective cancer cells inhibited metastasis formation. Additionally, the potential of colon cancer cells (HCT116) to form metastasis in vivo was abolished completely upon genetic deletion of PPARβ/δ. Treatment of mice with the PPARβ/δ agonist GW0742 enhanced metastasis formation. The metastatic potential of PPARβ/δ in cancer cells was confirmed in orthotopic tumor models, confirming that also spontaneous metastasis formation was dramatically reduced upon knockdown of PPARβ/δ. Using heterozygous PPARβ/δ mice for syngenic tumor cell vein injection the authors further demonstrated that high expression of PPARβ/δ in cancer cells is the most important factor for metastasis formation as heterozygous PPARβ/δ mice developed fewer metastasis than their wildtype littermates, but exhibited the most important reduction of metastasis formation when injected with PPARβ/δ knockdown cancer cells. Transcriptome profiling of HCT116 wildtype and PPARβ/δ knockout cells identified gap junction protein alpha 1 (GJA1), vimentin (VIM), secreted protein acidic rich in cysteine (SPARC), neuregulin-1 (NRG1), CXCL8 (IL-8), stanniocalcin-1 (STC1), and synuclein gamma (breast cancer-specific protein 1; SNCG) as pro-metastatic PPARβ/δ targets. Finally, the authors further confirmed the correlation of high PPARβ/δ expression and significantly reduced metastasis-free survival in various cancer patient (colorectal, lung, breast) cohorts, including the largest reported cohort of 1609 breast cancer patients [98].

In profound contrast to the extensive in vivo study of Zuo, Lim and coworkers reported increased melanoma cell migration and invasion upon treatment with the PPARβ/δ antagonist 10 h as well as increased metastasis formation in PPARβ/δ knockout mice [133]. This antagonist had so far not been used in other studies and results were not confirmed employing well established antagonists as GSK0660 or GSK3787. Conversely, Ham and colleagues demonstrated that activation of PPARβ/δ in highly metastatic melanoma cell lines provoked an upregulation of Snail, a decrease of E-cadherin, and a stimulation of migration and invasion, which could be reversed by knockdown of PPARβ/δ. PPARβ/δ therefore seems to promote the high metastatic potential of aggressive melanoma [134].

Our group confirmed pro-metastatic effects of PPARβ/δ activation. Syngenic subcutaneous LLC1 tumor cell implantation resulted in significantly increased lung and liver metastasis when animals received the PPARβ/δ agonist GW0742. Interestingly, we also observed increased spontaneous metastatic spreading in a model with inducible conditional vascular-specific overexpression of PPARβ/δ, indicating that the proangiogenic function of PPARβ/δ importantly contributes to metastatic tumor progression [14].

Recently, an elegant study demonstrated the implication of PPARβ/δ in the pro-metastatic effects of dietary fats in colorectal cancer. Activation of PPARβ/δ with GW501516 induces cancer stem-like cell (CSC) expansion and accelerates liver metastasis in vivo. Analysis of promoters of self-renewal regulatory factors such as Oct4, Nanog, Sox2, and KLF4 identified a PPAR responsive element in the Nanog promoter. Activation of PPARβ/δ with GW501516 increased whereas knockout of PPARβ/δ decreased Nanog expression. Colonic CSC expansion was shown to be induced by PPARβ/δ through direct induction of Nanog expression via binding to its promoter. Furthermore, knockdown of Nanog abolished PPARβ/δ stimulation of hepatic metastasis formation. Similar to the exposure to GW501516, a high fat diet induced expression of Nanog, accelerated tumor growth and liver metastasis formation and knockout of PPARβ/δ completely inhibited these effects. This identifies a novel PPARβ/δ-mediated mechanism responsible for the contribution of dietary fat to colorectal cancer initiation and metastasis [135].

In conclusion, overwhelming evidence suggests that PPARβ/δ promotes metastasis.

## 7. PPARβ/δ and Replicative Immortality

Activation of PPARβ/δ with GW501516 was shown to inhibit angiotensin (Ang) II induced premature senescence of human vascular smooth muscle cells (hVSMCs). Ang II treatment of hVSMCs provoked an increase of senescence associated beta galactosidase activity (SA β-gal), which was inhibited by GW501515, an effect that could be reversed by hVSMCs knockdown. A significant reduction of SA β-gal activity was also observed upon pretreatment with N-acetyl-l-cysteine (NAC), a thiol antioxidant, suggesting that reactive oxygen species (ROS) mediate Ang II-induced premature senescence of hVSMCs. Activation of hVSMCs significantly reduced ROS accumulation as well as DNA damage in hVSMCs treated with Ang II. PPARβ/δ mediated transcriptional up-regulation of antioxidant genes (glutathione peroxidase (GPx)-1, manganese superoxide dismutase (Mn-SOD), heme oxygenase (HO)-1, and Thioredoxin (Trx)-1) had been identified as the major mechanism in the inhibition of premature senescence of hVSMCs [136]. In a following study, the authors identified upregulation of phosphatase and tensin homolog deleted on chromosome 10 (PTEN), leading to suppression of phosphatidylinositol 3-kinase (PI3K)/Akt pathway, by PPARβ/δ as a second mechanism of senescence inhibition in hVSMCs [137]. Increase of PTEN and suppression of PI3K/Akt by PPARβ/δ activation was also the main pathway identified for senescence inhibition of UV-induced keratinocytes by the agonist GW501516 [138]. In human coronary artery endothelial cells, inhibition of Ang II induced senescence by PPARβ/δ was found to be dependent of transcriptional activation of Sirtuin (SIRT) 1. Downregulation or inhibition of SIRT1 abolished the effects of PPARβ/δ on Ang II induced ROS production and premature senescence, and resveratrol, a SIRT1 activator, mimicked PPARβ/δ agonist effects [139]. PPARβ/δ activation has also been shown to prevent doxorubicin induced cardiomyocyte senescence. The PPARβ/δ agonist L165041 prevented telomeric repeat factor (TRF) 2 downregulation, partially rescued cell proliferation blockage, significantly attenuated cytoskeletal remodeling and the early loss of plasma membrane integrity and significantly reduced SA-β-gal activity. Senescence inhibition was in this case shown to be dependent of B-cell lymphoma 6 protein (Bcl6) as a potent inhibitor of senescence, rendering cells unresponsive to antiproliferative signals from the p19ARF–p53 pathway. L1650141 increased the expression of Bcl6, which upon ligand binding, was released from PPARβ/δ and repressed its target genes, involved in DNA damage sensing and proliferation of checkpoint control [140]. In this context, it might be interesting to mention that our group observed an increase of TRF2, a protein that has a key role in the protective activity of telomeres [141], in tumor sorted endothelial cells from mice with vascular specific overexpression of PPARβ/δ (Wagner et al., unpublished results). 

In contrast to these studies reporting senescence inhibition upon PPARβ/δ activation, Zhu and coworkers observed stimulation of Harvey sarcoma ras virus gene (Hras)-induced senescence by PPARβ/δ. 7,12-dimethylbenz[a]anthracene (DMBA)-initiation led to a higher percentage of malignant squamous cell carcinomas and a lower percentage of benign papillomas in PPARβ/δ knockout compared to wildtype animals. In vitro, Hras expressing PPARβ/δ knockout keratinocytes displayed less senescence as investigated by SA β-gal staining. The authors identified as the molecular mechanisms of this senescence induction by PPARβ/δ a potentiation of the RAF/MEK/ERK pathway and an inhibition of the PI3K/AKT pathway [142]. In a very similar study appearing in the same year, the authors showed that increased endoplasmatic reticulum (ER) stress attenuated senescence in part by up-regulating phosphorylated protein kinase B (p-AKT) and decreasing phosphorylated extracellular signal-regulated kinase (p-ERK), which was repressed by PPARβ/δ [143].

Cellular senescence has been linked to the development of endothelial cell dysfunction in atherosclerosis. Especially oxidative stress induced by ROS from lipid loaded macrophage foam cells has been linked to premature senescence of the vasculature. Riahi and coworkers exposed endothelial cells to the secretome of such foam cells and observed an increase of endothelial SA β-gal activity, p16 and p21 expression as well as a decrease of phosphorylated retinoblastoma protein. They found that senescence was induced by 4-hydroxnonenal (4-HNE) through stimulation of pro-oxidant thioredoxin-interacting protein (TXNIP). The lipid peroxidation product 4-HNE activated PPARβ/δ promoter activity. The PPARβ/δ agonist GW501516 enhanced TXNIP expression, whereas the antagonist GSK0660 reduced TXNIP promoter activity and inhibited 4-HNE induced senescence [144].

In contrast to the prosenescent effects of PPARβ/δ in endothelial cells, Bernal and colleagues reported that PPARβ/δ maintains the proliferative undifferentiated phenotype of adult neuronal precursor cells, probably through activation of SOX2, one self-renewal regulatory factor [145]. This is in line with the findings from Wang and colleagues showing that colonic cancer stem cell expansion was induced by PPARβ/δ through direct transcriptional activation of Nanog [135]. 

It has been described that PPARβ/δ amplifies Wnt signaling activity through direct interaction with β-catenin and direct transcriptional activation of the Wnt coreceptor low-density lipoprotein receptor-related protein (LRP) 5 [146]. Senescence associated reprogramming has been shown to upregulate an adult tissue stem-cell signature in lymphoma cells, activate Wnt signaling and distinct stem-cell markers. Former senescent lymphoma cells had a higher in vivo tumor initiation potential than their non-senescent counterparts [147]. Given these highly interesting findings, it will be extremely exciting to further clarify the role of PPARβ/δ in cancer related senescence, replicative immortality and cancer stemness.

## 8. PPARβ/δ and Metabolism

It is well established that the high tumor cell growth rate due to proliferation is connected to profound metabolic changes [12]. As early as 1927, Otto Warburg described an anomaly in cancer cell metabolism compared to normal cells—cancer cells largely depend on aerobic glycolysis for energy production [148,149,150]. Cancer metabolism is not only linked to proliferation, but also to tumor angiogenesis as rapidly growing tumor cells will turn on the “angiogenic switch” for increased oxygen supply in the tissue. Lack of oxygen results in hypoxia in the tissue, which results in stabilization of hypoxia- induced factors (Hif) [151,152,153] and subsequent activation/inhibition of downstream target genes, e.g., VEGF [154], erythropoietin [155], WT1 [156], PPARα [157], glucose transporters (Glut-1 and Glut-3) and many other target genes involved in cancer metabolism (for a recent review see [158]). In contrast to PPARα, PPARβ/δ seems not to be directly regulated by Hif-1; but Hif-1 expression is stimulated by calcineurin A [159] and PPARβ/δ activates calcineurin [85]. Consequently, we observed an increase in calcineurin and Hif-1 expression in the hearts of mice treated with the PPARβ/δ agonists GW0742 and GW501516 [85]. Whether this signaling cascade is relevant for PPARβ/δ-dependent cancer progression remains to be established. Hypoxic stress has been shown to induce transcriptional activation of PPARβ/δ in HCT116 colon cancer cells. PPARβ/δ associated with p300 upon hypoxic stress in these cells. The p300 and the PI3K/Akt pathways seem to play a role in the regulation of PPARβ/δ transactivation as PI3K inhibitors or siRNA knockdown of Akt suppressed the PPARβ/δ transactivation in response to hypoxia [160]. Interestingly, hypoxia-induced IL-8 and VEGF expression was significantly attenuated in PPARβ/δ-deficient colon cancer cells linking expression of PPARβ/δ in cancer cells to tumor angiogenesis and immune response [160]. The in vivo relevance of these findings for tumor growth remains to be determined. In addition, prostacyclin synthase, which catalyzes the conversion of prostaglandin H2 (PGH2) to prostaglandin I2 (PGI2) is upregulated in fibroblasts and cancer cells in response to hypoxia. PGI2 in turn stimulates PPARβ/δ and subsequent VEGF expression [161], which provides an additional link between hypoxia, metabolism, PPARβ/δ in the tumor stroma and angiogenesis. PPARβ/δ also protects chronic lymphocytic leukemia and breast cancer cells from harsh environmental conditions, i.e., hypoxia and low glucose concentrations, which was related to increased antioxidant expression, substrate utilization and mitochondrial performance providing additional evidence for PPARβ/δ as a positive regulator of cancer growth [28,35].

Long chain fatty acids (LCFA) represent energy sources, components of cell membranes and are further processed into signaling molecules. Dietary fatty acids are linked to cancer risk especially colon cancer. Saturated fatty acids were positively associated with colon cancer risk, while polyunsaturated fatty acids showed inverse associations [162]. Experimental studies, however suggested that saturated long chain fatty acids (SLCFA) inhibit while unsaturated long chain fatty acids (ULCFA) might increase proliferation of different cancer cell lines [163,164]. A recent report provided novel mechanistic insights into this problem linking long chain fatty acid metabolism and cancer [165]. Saturated fatty acids bind to fatty acid binding protein 5 (FABP5) and displace endogenous ligands and retinoic acid (RA) from this transport protein. Thus, these ligands are not delivered to PPARβ/δ and its transcriptional activity is reduced while RA is diverted to the retinoic acid receptor (RAR), which becomes activated. In contrast, binding of unsaturated long-chain fatty acids to FABP5 has similar consequences for the displacement of RA and its subsequent binding to RARs, but results in nuclear import of the ULCFA/FABP5 complex and subsequent activation of PPARβ/δ, which in turn results in increased cancer cell proliferation [165]. Although these results identify a central role for FABP5 for cancer cell proliferation and might explain the differences observed regarding PPARβ/δ and cancer cell proliferation dependent on the presence/absence of FABP5 and amounts of RA and endogenous PPAR ligands, the situation for in vivo experimental and clinical studies might be even more complex due to the interplay of the different hallmark capabilities.

PPARβ/δ is, however, not only activated by fatty acids presented by FABP5 in tumorigenesis. In mammary epithelium, overexpression of PDK1 resulted in increased phosphorylation of Akt and GSK3β and augmented expression of PPARβ/δ protein. Treatment with GW501516 increased the number of mammary tumors and reduced survival, which was even more pronounced in animals with PDK1 overexpression. This dramatic effect correlated with an increase in a specific metabolic gene signature indicative of glycolysis and greater levels of fatty acid and phospholipid metabolites in PDK1 overexpressing mice treated with GW501516 compared to treated wild-type control mice [38]. As these metabolic changes are common also in human tumors [166] and enable high tumor cell proliferation [167], it is possible that this mechanism plays a common role in tumor types with PPARβ/δ overexpression. In addition, GW501516 increases expression of glucose transporter 1 (Glut-1) and solute carrier family 1 member 5 (SLC1A5), which results in an increased influx of glucose and glutamine in different cancer cell types and subsequently augments cancer cell proliferation [15]. Furthermore, animals with direct specific overexpression of PPARβ/δ in the mammary epithelium were prone to the development of mammary tumors [36]. Infiltrating mammary ductal carcinomas developed after a latency of 12 months; GW501516 reduced tumor latency to 5 months. Histologically, PPARβ/δ overexpression was confirmed in the mammary epithelium. In agreement with the study by Pollock et al. [38], increased Akt phosphorylation was detected, but also mTOR was activated. Inhibition of mTOR by everolimus reduced cell proliferation and the malignant phenotype indicating the importance of this signaling pathway for PPARβ/δ-dependent mammary tumorigenesis. Microarray and metabolomic analyses revealed a marked increase in the levels of phosphatidylcholine metabolites, lysophosphatidylcholine, lysophosphatidic acid and arachidonic acid metabolites, which correspond to PPARβ/δ-dependent gene regulation involved in prostaglandin biosynthesis. Lysophosphatidic acid stimulated mTOR activation through Akt, and phosphatidic acid directly mediates activation of mTOR [36]. These results provided robust evidence for PPARβ/δ induced metabolic changes resulting in mTOR activation in mammary tumorigenesis. Taken together, several metabolites increase PPARβ/δ activity and PPARβ/δ stimulation induces complex metabolic alterations, which are mostly protumorigenic.

## 9. PPARβ/δ and Immune Function

PPARβ/δ agonists have been reported to inhibit the tumor necrosis factor (TNF) α induced up-regulation of monocyte chemoattractant protein (MCP)-1 and vascular cell adhesion protein (VCAM)-1 in endothelial cells, to inhibit cytokine induced nuclear translocation of NF-kappaB and to reduce monocyte binding to activated vascular cells [74]. They modulate acute inflammation by targeting the neutrophil-endothelial cell interaction and reducing tumor necrosis factor alpha induced endothelial chemokine ligand (CXCL) 1 release and VCAM-1, E-selectin and ICAM-1 expression [77]. Another study described potent inhibitory effects of the PPARβ/δ agonist GW0742 on lipopolysaccharide target genes as cyclooxygenase (COX)-2 and inducible nitric oxide synthase (iNOS) in macrophages. Lipopolysaccharide (LPS) is the most abundant component within the cell wall of Gram-negative bacteria. It can stimulate the release of inflammatory cytokines in various cell types, leading to an acute inflammatory response towards pathogens. It has been suggested that PPARβ/δ functions in modulating the program of macrophages during inflammatory responses [168]. PPARβ/δ modulation has been proposed to attenuate inflammation in atherosclerosis. A comparison between wildtype and PPARβ/δ knockout macrophages revealed that proinflammatory genes such as MMP9, IL-1β and MCP-1 were down-regulated in PPARβ/δ knockout macrophages. However, activation of PPARβ/δ with GW501516 suppressed the expression of MCP-1 and IL-1β, indicating that activation of PPARβ/δ is anti-inflammatory. As an explanation for this seemingly discrepancy of PPARβ/δ function in inflammation, a ligand-dependent interaction of PPARβ/δ with the anti-inflammatory transcriptional repressor BCL-6 had been suggested. Without ligand, PPARβ/δ binds BCL-6. When activated with a PPARβ/δ ligand, BCL-6 is released and suppresses proinflammatory pathways [75]. Monocytes can be differentiated in either a proinflammatory (M1 or classically activated macrophage, induced by TNFα, bacterial LPS or interferon gamma) or an anti-inflammatory (M2 or alternatively activated macrophage, induced by interleukins). PPARβ/δ has an important role in the development of the M2 phenotype, as PPARβ/δ knockout cells were unable to acquire this alternatively activated macrophage phenotype upon interleukin-4 or-10 stimulation [169]. In contrast, Thulin and colleagues demonstrated that PPARβ/δ is regulated by the microRNA miR-9 in monocytes and that activation of PPARβ/δ might be of importance in M1 proinflammatory, but not in M2 anti-inflammatory macrophages, as the PPARβ/δ agonist GW501516 induced expression of PPARβ/δ target genes in proinflammatory M1, but not in M2 macrophages [170]. Further studies confirmed the implication of PPARβ/δ in the modification of macrophage functions and the reprogramming of their activation status. Treatment of macrophages with modified low-density lipoproteins (LDLs) induced arginase I expression, which was abolished by the PPARβ/δ antagonist GW9662. In contrast, the PPARβ/δ agonist GW0742 strongly induced arginase I expression. PPARβ/δ activity in macrophages therefore impacts the balance of Th1/Th2 responses through specific induction of arginase I expression and activity [171]. Myelin-derived phosphatidylserine was found to mediate PPARβ/δ activation in macrophages after myelin uptake, a pathway leading to suppression of the production of inflammatory mediators, ameliorating experimental autoimmune encephalomyelitis (EAE), an animal model of multiple sclerosis [172]. Mukundan et al. identified PPARβ/δ as a transcriptional sensor of apoptotic cells in macrophages. Apoptotic cell feeding stimulated PPARβ/δ expression in macrophages, which then induced expression of opsonins, enhanced apoptotic cell clearance by macrophages and increased anti-inflammatory cytokine production [173]. As another mechanism of PPARβ/δ function in macrophages, induction of the immunoreceptor CD300a has been postulated. The PPARβ/δ agonist GW501516 activated CD300a expression in macrophages. Mice lacking CD300a showed chronic intestinal inflammation upon high fat diet and an increase in proinflammatory cytokines, specific for the M1 macrophage type. The PPARβ/δ/CD300a pathway could therefore contribute to the anti-inflammatory action in macrophages [174]. Adhikary and colleagues investigated the global PPARβ/δ-regulated signaling network in human monocyte-derived macrophages. They found a robust induction of PPARβ/δ expression upon monocyte to macrophage differentiation. Using PPARβ/δ agonists and inverse agonists, they identified two mechanisms by which PPARβ/δ regulates immune-modulatory genes: 1) canonical regulation through DNA binding at PPARβ/δ–RXR sites (PPREs), induced by agonists and repressed by inverse agonists, and 2) repression by agonists in the absence of PPARβ/δ DNA binding (inverse regulation). Inverse regulation concerned NF-kappaB and the signal transducer and activator of transcription (STAT)1 target genes, resulting in the inhibition of multiple proinflammatory mediators consistent with anti-inflammatory effects of PPARβ/δ activation. Interestingly, they could also demonstrate specific immune stimulatory effects induced by PPARβ/δ agonists, a pro-survival effect on macrophages and inhibition of CD32B surface expression and stimulation of T cell activation. This confirms the strong anti-inflammatory function of PPARβ/δ, but also indicates context-dependent specific immune-stimulatory actions of PPARβ/δ activation [175]. The same group aimed at elucidating the role of PPARβ/δ in the pro-tumorigenic polarization of tumor associated macrophages (TAMs) in ovarian cancer. In vitro, PPARβ/δ target genes such as pyruvate dehydrogenase kinase (PDK) 4 and angiopoietin-like protein (ANGPTL) 4 were robustly induced in monocyte derived macrophages, but the ligand response in TAMs was impaired and most PPARβ/δ target genes were refractory to synthetic agonists. Next, the authors compared freshly isolated ascites-associated TAMs from ovarian cancer patients with monocyte-derived macrophages from healthy donors. Many PPARβ/δ target genes as PDK4, ANGPTL4, and carnitine palmitoyl transferase (CPT) 1A were found to be up-regulated in TAMs and were refractory to stimulation with the PPARβ/δ agonist L-165041. The deregulation and unresponsiveness of target genes in TAMs was found to be due to the presence of endogenous activators in malignancy associated ascites, as ascites caused an equal deregulation in normal macrophages. Lipidomic analysis of ascites samples revealed high levels of polyunsaturated fatty acids (PUFA) [176], known as PPARβ/δ activators [177]. The deregulation of PPARβ/δ target genes by PUFA ligands stimulates the pro-tumorigenic conversion of host-derived monocytic cells and might contribute to tumor progression [176]. Very little is known about the PPARβ/δ function in other key immune cell types except macrophages. In 2008, protein expression of PPARβ/δ in activated human T-cells was described. It has been shown that PPARβ/δ is a transcriptional target of human type I interferon (IFN), stimulates T-cell proliferation and inhibits IFN induced apoptosis, which is partially mediated through enhanced extracellular signal-regulated kinases (ERK) 1/2 signaling [178]. More recently, it has been demonstrated that PPARβ/δ overexpression/activation in vivo inhibits thymic T-cell development by decreasing proliferation of CD4^-^CD8^-^ double-negative stage 4 (DN4) thymocytes [179]. PPARβ/δ has further been reported to drive maturation of monocyte–derived dendritic cells towards an atypical phenotype with reduced stimulatory effects on T-cells [180]. An interesting in vivo study using murine models of septic shock induction confirmed general anti-inflammatory effects of PPARβ/δ activation. PPARβ/δ deletion had detrimental effects on cardiac and renal function, liver injury, lung inflammation and survival, which could not be attenuated by administration of the specific agonist PPARβ/δ GW0742. In wildtype animals, selective activation of PPARβ/δ attenuated the multiple organ injury and dysfunction and improved survival when administered acutely in rodent models of endotoxemia and polymicrobial sepsis. PPARβ/δ activation was proposed as an anti-inflammatory therapeutic approach for the treatment of conditions involving local and systemic inflammation [181]. Using an experimental model for multiple sclerosis, it has been shown that PPARβ/δ limits the expansion of pathogenic T helper cells and production of Interleukin 12 and Interferon gamma, thereby limiting autoinflammation in the central nervous system [182]. Similar, in acute cerulein and taurocholate induced pancreatitis mouse models, treatment with the PPARβ/δ agonist GW0742 reduced expression of proinflammatory enzymes and cytokines, neutrophil invasion and tissue and inflammatory deterioration in the pancreas [183]. In contrast, PPARβ/δ has been shown to be a negative regulator of mesenchymal stem cell (MSC) immunosuppressive function, as PPARβ/δ inhibition or genetic deletion enhanced the immunosuppressive properties of MSCs, involving an increased NF-kappaB, ICAM-1 and VCAM-1 activity [184]. Interestingly, also in natural killer (NK) cells, inhibition of PPARβ/δ was beneficial to restore cytotoxic anti-tumor activity. Obesity induced a PPAR driven lipid accumulation in NK cells causing inhibition of their cellular metabolism and inhibiting their function. PPARβ/δ agonists mimicked obesity effects and inhibited trafficking of the cytotoxic machinery to the NK cell-tumor junction, disenabling NK cells to reduce tumor growth in obesity in vivo. Inhibition of PPARβ/δ restored NK cell cytotoxicity [185]. Finally, it may be concluded that most studies identified PPARβ/δ function as anti-inflammatory, mainly in the setting of atherosclerosis. However, only few cancer related investigations exist. In this context, PPARβ/δ has pro-inflammatory and pro-tumorigenic functions by converting host monocytes in macrophages favoring tumor progression [176,185] or impairing antitumor cytotoxicity of NK cells. Surely, more cancer related studies addressing the question how PPARβ/δ acts in different immune regulatory cells, tissues and conditions, are needed.

## 10. Conclusions and Outlook

PPARβ/δ functions have been studied extensively. We summarized here known PPARβ/δ effects on cell proliferation, induction of angiogenesis, cell death, function of tumor suppressors, replicative immortality and senescence, invasion and metastasis, tumor metabolism and immune function and mentioned underlying molecular mechanisms. Although not all cited manuscripts were directly related to cancer, one has to keep in mind that the different hallmark capabilities interplay during tumor progression [12,13]. Some controversies regarding the effects of PPARβ/δ activation for cancer progression still exist, which might relate to the different cellular or animal models used. The majority of reports, however, suggest that activation of PPARβ/δ might result in modifications of the hallmark capabilities in favor of a pro-tumorigenic profile. Thus, in contrast to the earlier notion of the therapeutic potential of PPARβ/δ agonists as “exercise mimetics” and potential treatments for metabolic syndrome [186,187,188], extreme caution should be applied when considering PPARβ/δ agonists for therapeutic purposes given their pro-tumorigenic properties. 

For future approaches using PPARβ/δ modulation for potential cancer therapy, collaborations between different laboratories and pathologists are urgently needed to define exact expression patterns of PPARβ/δ in different types, stages and grades of cancer. Currently, already the antibody validation is a limiting factor. Reproducible immunostaining protocols established between different laboratories and precise annotation of cell types would be required to define, which patients might benefit from PPARβ/δ modulation according to expression pattern in cells of the different hallmark capabilities.

## Figures and Tables

**Table 1:** Effects of PPARβ/δ on cell proliferation and tumor growth.

Model	Intervention	Outcome	Reference
Wild-type mice	GW0742 agonist	LLC1 tumor growth ↑, Metastasis ↑	[14]
Endothelial-specific PPARβ/δ overexpression	LLC1 tumor induction	Tumor growth ↑, Metastasis ↑	[14]
Nude Mice, SW480 cells	GW501560 GW501516 + Metformin	Tumor growth ↑ Tumor growth ↓	[15]
Apc(Min/+) mice PPARδ^−/−^/Apc^Min/+^ mice	GW501560 GW501560	Tumor growth ↑ Tumor growth ↓	[8,16]
Apc^Δ^^580^ mice Apc^Δ^^580^ mice	GW501560 GSK3787	Tumor growth ↑ Tumor growth ↓	[7,17]
Wild-type mice colitis model	GW501560	Epithelial cell proliferation ↑, Tumor growth ↑	[18]
Colitis-associated colon cancer mice	PPARβ/δ overexpression	Tumor growth ↑	[19,20]
Several mouse models	High Fat diet, GW501516	Intestinal stem and progenitor cell proliferation ↑, Tumor growth ↑	[21]
Azoxymethane-induced colon tumors	Colon-specific PPARβ/δ knockout	Tumor growth ↓	[22]
Azoxymethane-induced colon tumors	GW0742	Tumor growth ↓	[23]
Nude mice with KM12C colon cells	PPARβ/δ silencing	Tumor growth ↑	[24]
PPARβ/δ- floxed mice	Prostate-specific knockout	Cellularity ↑	[25]
Nude mice with DU145 prostate cancer cells	PPARβ/δ silencing	Cell number ↓, Tumor growth ↓	[26]
PC3M prostate cancer cells	GW0742 PPARβ/δ silencing	Cell number ↑, Cell number ↓	[27]
Daudi CLL cells	PPARβ/δ overexpression	Cell number ↑	[28]
Neuroblastoma cell lines	PPARβ/δ overexpression	Cell number ↓ in NGP, Cell number ≈ SK-N-BE(2) and IMR-32	[29]
Transgenic hepatitis B virus (HBV) mice	GW0742	Hepatic tumor foci ↓	[30]
Hepatocellular carcinoma cell lines	GW501560, PPARβ/δ RNAi	Proliferation ↑, Proliferation ↓	[31]
Melanoma cell lines	GW0742, GW501560	Proliferation ↓	[32]
UACC903 melanoma cells	GW0742, GW501560	Proliferation ↓	[33]
MCF-7, MDA-MB-231 breast cancer cell lines	GW501560	Proliferation ↓, Proliferation ≈	[34]
NOD-SCID mice with MCF-7 breast cancer cells	PPARβ/δ overexpression	Tumor growth ↑, Lung metastasis ↑	[35]
MCF-7 cells with PPARβ/δ overexpression	DG172, NXT1511 antagonists	Cell number ↓	[35]
PPARβ/δ overexpression in the mammary gland	GW501516	Spontaneous mammary carcinomas after of 12 months, after 5 months with agonist treatment	[36]
Cox2 overexpressing mice	PPARβ/δ knockout	Proliferation ↓, Tumor growth ↓	[37]
PDK1 overexpression in the mammary gland	GW501516	Tumor growth ↑	[38]
MMTV-ErbB2/HER2 onco-mice	FABP5 knockout	Tumor growth ↓	[39]
Mouse mammary tumorigenesis	GW501516	Tumor growth ↑	[40]
Testicular embryonal carcinoma cell lines	PPARβ/δ overexpression, GW0742	Tumor growth ↓, Proliferation ↓	[41]
PPARβ/δ-null mice	Chronic UV exposure	Tumor growth ↓	[42]
Non-small cell lung cancer cell lines	PPARβ/δ agonists PPARβ/δ silencing	Proliferation ↑ Proliferation ↓	[43]
Lung cancer cell lines	PPARβ/δ agonists	Proliferation ≈	[44]
RAF-induced lung adenoma	PPARβ/δ knockout	Tumor growth ↑	[45]
Liposarcoma cell lines	PPARβ/δ agonists PPARβ/δ silencing	Proliferation ↑ Proliferation ↓	[46]
Primary human thyroid cells	PPARβ/δ overexpression, GW501516	Proliferation ↑	[47]
Epithelial ovarian cancer cell lines	Dominant negative PPARβ/δ	Proliferation ↓	[48]

↑ Increase; ↓ decrease; ≈ not significantly different; CLL: chronic lymphocytic leukemia; Apc: Familial Adenomatous Polyposis gene mutated; GW501516, GW0742—specific PPARβ/δ agonists; DG172, NXT1511, GSK3787— PPARβ/δ antagonists.
